# 2750. *Pseudomonas aeruginosa* infections treated with cefiderocol: associations of site of infection and time to first dose with outcomes in PROVE (Retrospective Cefiderocol Chart Review) Study

**DOI:** 10.1093/ofid/ofad500.2361

**Published:** 2023-11-27

**Authors:** Stephen Marcella, Bin Cai, Bruce Friedman, Stefano Verardi, Laurence Gozalo, Christine M Slover

**Affiliations:** Shionogi Inc, Florham Park, New Jersey; Shionogi Inc, Florham Park, New Jersey; Joseph M. Still Burn Center, Augusta, Georgia; Shionogi, B.V., London, England, United Kingdom; Genesis Research, Hoboken, New Jersey; Shionogi Inc., Florham Park, New Jersey

## Abstract

**Background:**

Gram-negative (GN) bacterial resistance is a major global health problem. Cefiderocol (CFDC) is active against *Pseudomonas aeruginosa* (PA). PROVE is an international, retrospective study of CFDC use in Gram-negative infections (GNI). This analysis examines outcomes by infection site and time of first CFDC dose from culture for patients with PA as of March 2023.

**Methods:**

Eligible patients received ≥ 72 hours of CFDC as used in routine clinical practice and had a PA culture attributed to the use of CFDC. Patient characteristics, clinical course severity, and treatment patterns were assessed. Associations of infection site, carbapenem resistance (CR), CFDC susceptibility, and time of first (index) culture sample to first CFDC dose were examined with clinical cure and all-cause mortality (ACM) at 30 days. Clinical cure at the end of treatment was defined as resolution or improvement in infection signs and symptoms without evidence of later relapse.

**Results:**

253 patients were treated with CFDC at 39 sites in the US and Europe for a primary PA infection; 30.8% were polymicrobial with other GN pathogens (Table 1). The median age was 57 years, interquartile range (IQR) 45-65; 70% were male. The most frequent comorbidities were diabetes (34.0%) and chronic pulmonary disease (20.2%). CFDC was given in an ICU setting in 72.3% with 48.6% requiring mechanical ventilation.

The most common sites of infection were respiratory (n=166, 65.6%) of which 22 (15.3%) had secondary bacteremia (Table 1). Most (90.3%) were CR. The median time from a positive culture to start of CFDC was 5 days (IQR 3-8).

Clinical cure was 63.6% and 30-day ACM was 22.5% (Table 1). Clinical cure was comparable in respiratory with and without secondary bacteremia (63.2% and 68.2%, respectively) and was lowest in patients with skin structure infections with bacteremia (40.0%). CFDC started 3-4 days after culture had the greatest cure rate (70.8%); those started later (5-7 days) had the lowest (59.2%). 30-day ACM was also greatest for patients treated later ( > 7 days after index culture) (28.1%) and for salvage after prior treatment failure (26.7%) (Table 2).

**Figure:**
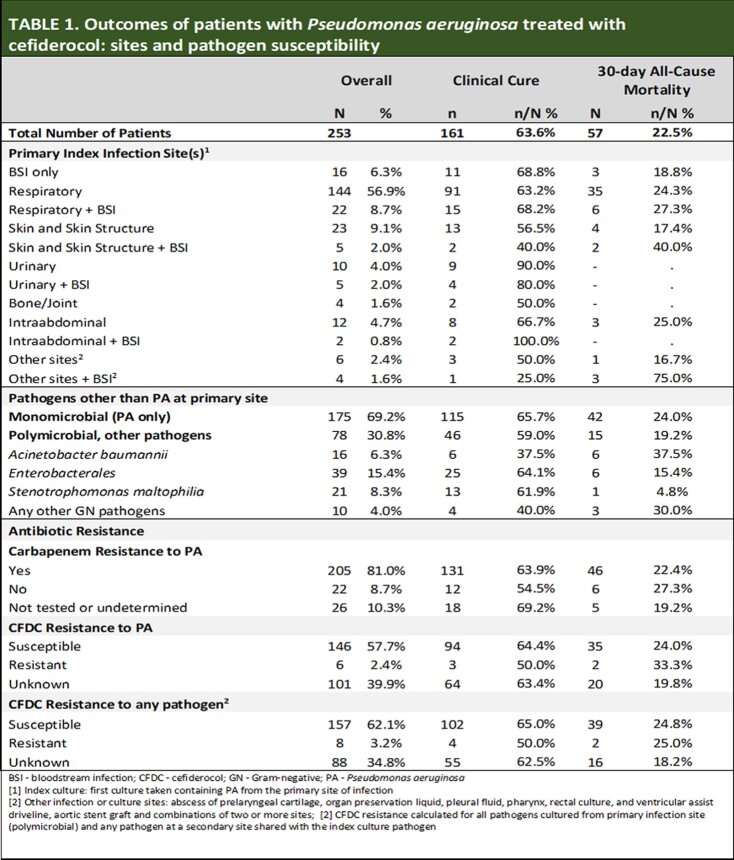

**Figure:**
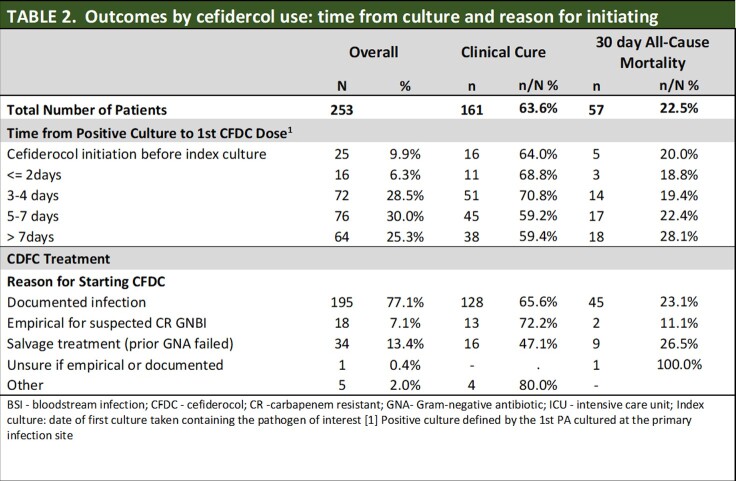

**Conclusion:**

CFDC for PA is used in complex patients, most with respiratory disease. The trend of better outcomes with earlier use from culture warrants additional study.

**Disclosures:**

**Stephen Marcella, MD, MPH**, Shionogi, Inc: contracting work for Shionogi, Inc **Bin Cai, MD, PhD**, Shionogi Inc.: Shionogi employee **Bruce Friedman, MD**, Shionogi: Honoraria **Stefano Verardi, MD**, Shionogi B.V.: Employee **Laurence Gozalo, PhD**, Shionogi, Inc: Analyst **Christine M. Slover, PharmD**, Shionogi,INC: Employee

